# The red-beard evolutionary explanation of human sociality

**DOI:** 10.1007/s40656-021-00471-x

**Published:** 2021-11-22

**Authors:** Vaios Koliofotis

**Affiliations:** Erasmus Institute for Philosophy and Economics, Erasmus University Rotterdam, Burgemeester Oudlaan 50, 3062 PA Rotterdam, Netherlands

**Keywords:** Altruism, Cooperation, Emotion, Evolution, Green-beard, Signal

## Abstract

Recent evolutionary studies on cooperation devote specific attention to non-verbal expressions of emotions. In this paper, I examine Robert Frank’s popular attempt to explain emotions, non-verbal markers and social behaviours. Following this line of work, I focus on the green-beard explanation of social behaviours. In response to the criticisms raised against this controversial ultimate explanation, based on resources found in Frank’s work, I propose an alternative red-beard explanation of human sociality. The red-beard explanation explains the emergence and evolution of emotions, a proximate cause, rather than patterns of behaviour. In contrast to simple evolutionary models that invoke a green-beard mechanism, I demonstrate that the red-beard explanation can be evolutionary stable. Social emotions are a common cause of a social behaviour and a phenotypic marker and therefore cooperative behaviour cannot be suppressed without also changing the marker.

## Introduction

Emotion-based communication is a key feature of our daily life, with human interactions being replete with many forms of verbal and non-verbal emotional expressions. Verbal exchanges include speech intonation and the use of language while non-verbal interactions consist of gestures, bodily postures, facial expressions, blushing or perspiration used to convey emotional states. Both verbal and non-verbal expressions of emotions appear to facilitate communication in the social world by providing quick information to others.

Non-verbal displays of emotions have been extensively studied by disciplines such as psychology, neuroscience, economics and biology. Darwinian evolution is widely acknowledged as a key for understanding human emotions. Since Charles Darwin’s classic book “The Expression of the Emotions in Man and Animals” (Darwin, 1872), evolutionary research on emotions was mostly concerned with identifying and characterizing non-verbal manifestations of discrete emotional states (e.g. Ekman, 1993; Izard, 2010). Joy, fear, surprise, anger, sadness and disgust^1^ are considered evolved human characteristics expressed by particular facial expressions across human groups.

More recently, evolutionary theorists systematically link evolutionary explanations of human non-verbal emotional expressions to animal signalling theory (e.g. Dezecache et al., 2013; McCullough & Reed, 2016). Moreover, the theoretical study of the biological evolution of social behaviours or social evolution theory examines phenotypic markers that facilitate assortment and the evolution of cooperation (e.g. Frank, 1987, 1988; Nesse, 2001; Riolo et al., 2001). This work has incited a recent trend in empirical research that investigates whether smiles (e.g. Reed et al., 2012; Centorrino, Djemai, Hpfensitz, Milinki, & Seabright, 2015), tears (e.g. Gračanin, Bylsma, & Vingerhoets, 2018; Reed et al., 2019), blushing faces (e.g. Dijk et al., 2011) and expressions of anger (e.g. Reed et al., 2014) are associated with social behaviours. Philosophical research in evolutionary ethics often relies on Robert Frank’s older theorizing on social emotions like love, anger, sympathy or jealousy and social behaviours (e.g. Heath & Rioux, 2018; James, 2011; Joyce, 2006).

Following this line of work, I discuss recent attempts to explain the evolution of emotional markers and social behaviours. Despite the diversity of evolutionary models in social evolution theory and animal signalling theory, even a cursory look at this literature reveals that a certain issue arises again and again in different contexts. At the theoretical level, many of these evolutionary studies rely on a simple but controversial green-beard complex to explain the evolution of altruistic cooperation (e.g. Cohen, 2012; Gardner & West, 2009; Handfield et al., 2018; West & Gardner, 2010). Evolutionary theorists have long emphasized that the green-beard explanation of social behaviours are inherently unstable or short lived. Without opportunities for reciprocation, a correlation between marker and social behaviour can be disrupted by the spread of a mutant gene, which adopts the phenotypic marker of cooperative individuals and elicits a favorable behavioural response.

In what follows I will not review or evaluate empirical research and experiments that test hypotheses about different emotional expressions^2^. It is also beyond the scope of this paper to present different theories in evolutionary ethics. Instead, I focus on Robert Frank’s account (Frank, 1987, 1988, 2005) and the arguments raised against it (e.g. Gardner & West, 2009; Heath & Rioux, 2018). My goal is to strengthen the case for the value of Frank’s theoretical work in explaining the evolution of human sociality.

In particular, I argue that recent well put criticism against Frank’s account of social behaviour does not render his ideas useless. Using resources found in Frank’s work, I distinguish between the red-beard evolutionary explanation that targets proximate causes such as emotions that govern behaviour and phenotypic markers and the green-beard explanation provided by Robert Frank’s evolutionary model that focuses on behaviour and fitness outcomes. Once we distinguish between these two different kinds of evolutionary explanation, it transpires that we do not need to abandon the idea of evolutionary stable communication between individuals based on emotions. In the red-beard explanation there a causal link between human emotions, social behaviour and phenotypic markers and this makes a substantial difference in the way one addresses the stability problem. Social emotions are a common cause of a social behaviour and a phenotypic marker and therefore cooperative behaviour cannot be suppressed without also changing the marker. Hence, the red-beard explanation is more plausible than it might appear at first glance.

The structure of the paper is as follows. In the next section I present the green-beard explanation of cooperation. Section 3 examines conceptual issues related to Robert Frank’s evolutionary model. Section 4 distinguishes between the red-beard and the green-beard explanations of cooperation and provides a solution to the stability problem. Section 5 concludes.

## The inclusive fitness analysis of human sociality

In this section I examine ultimate explanations of social behaviours that rely on the green-beard complex. The advantage of doing so is that it allows us to compare explanations as they are provided by different theoretical accounts and focus on what makes evolutionary explanations in the social sciences different in kind.

Social evolution theory is based on the ultimate-proximate distinction (Mayr, 1961). According to Vromen (2017), there are at least three ways to view Mayr’s distinction: First, there is a distinction between evolutionary causes (processes such as natural selection) and proximate behaviour-generating causes (e.g. psychological, neurological) inside organisms. Second, there is a distinction between distant and nearby parts in the causal chain that lead up to a behaviour. Finally, there is a distinction between different explanatory projects: ultimate explanations typically answer why questions (why did social behaviour evolve?) while proximate explanations typically answer how questions (how is social behaviour produced?).

Based on this insight, Vromen explicates the conventional view to answering ultimate why-questions in social evolution theory. Ultimate explanations examine why behaviours evolved while proximate explanations how they work. For example, if a phenotypic marker is explained in terms of intentional decision-making and behaviour-generating motives, researchers provide a proximate explanation^3^. If the same phenotypic marker is explained in terms of the dynamic of natural selection and how, on average, it has positive fitness consequences they provide an ultimate explanation. It is generally accepted that ultimate and proximate explanations complement each other and both are required for a complete account of social behaviour.

In social evolution theory behaviours are typically classified based on different combinations of their positive or negative effect on the actor and others’ fitness (e.g. Hamilton, 1964; West et al., 2007). In particular, a behaviour that is beneficial to the actor and is costly to the recipient is selfish. Altruistic behaviour is costly to the actor and beneficial to the recipient while mutually beneficial behaviour is beneficial both to the actor and the recipient’s fitness. Finally, a behaviour that is costly in terms of direct fitness to both the actor and the recipient is spiteful. Mutually beneficial and altruistic behaviours are often referred as cooperative behaviours.

The challenge in social evolution theory is to explain why social behaviours like altruism could have evolved despite being costly to the organisms that perform them. This puzzle has received a great deal of attention and generated a voluminous body of literature. The solution relies on the idea that the evolution of social behaviours is driven by effects on a focal individual’s own direct fitness and effects on the fitness of individuals that have the same gene(s) (i.e. indirect fitness). Based on this distinction, ultimate explanations of altruism and mutually beneficial behaviours are classified into two broad categories: Direct and indirect ultimate explanations. In direct fitness explanations social behaviour evolves because of an increase in the direct fitness of the actor. For example, in repeated interactions (Trivers, 1971; Axelrod & Hamilton, 1981) or collaboration (Sterelny, 2016) the actor might gain a delayed direct fitness benefit that is higher than the immediate fitness cost. By contrast, indirect fitness explanations explore the presence of genetic relatedness and indirect fitness benefits. Social behaviour evolves because indirect fitness is higher than the direct fitness cost for the actor.

One can further distinguish indirect ultimate explanations of social behaviour based on how fitness benefits arise in interactions between individuals that share the same gene(s). There are explanations that rely on genealogical kinship and green beard explanations based on a linkage or pleiotropy that allows the actor to gain an indirect fitness benefit (Dawkins, 1976; Hamilton, 1964). In what follows I focus on the green-beard explanation of cooperation that is relevant to the discussion of emotional markers in the sections that follow.

In the original formulation of the green-beard explanation, Hamilton (1964) considered a specific pleiotropy, a "supergene affecting (a) some perceptible feature of the organism, (b) the perception of that feature, and (c) the social response consequent upon what was perceived" (Hamilton, 1964, pp.25). The supergene hypothesis is explicit about the underlying genetics: one gene influences a set of phenotypic traits. It simultaneously encodes for a social behavioural response, a conspicuous phenotypic marker and the capacity to recognize this feature of an organism.

Dawkins analyzed Hamilton’s explanation and coined the widely used term “green-beard” to describe Hamilton’s perceptible feature of the “supergene”. A single gene might produce a distinct recognizable marker (i.e. the green-beard)^4^ and a tendency to behave in a “nice way” to those that carry that trait (Dawkins, 1976, pp.96). Although it is often unclear what kind of social behaviour is part of the green-beard complex, it is typically assumed that the green-beard explanation introduced by Hamilton and Dawkins refers to altruistic behaviour (Dawkins, 1982, pp.145). Bearers of a phenotypic marker (i.e. the green-beard) incur a fitness cost to provide direct fitness benefits to those that also have a green-beard. This type of social behaviour is thus personally costly to that particular altruist in direct fitness terms, but linkage or pleiotropy result to an increase in indirect fitness and allows altruistic behaviour to evolve.

More recent formulations of the greenbeard explanation refer to cases that involve distinct but tightly linked variant gene forms (i.e. alleles) that encode for the visible phenotypic markers and altruistic behaviour (e.g. Biernaskie et al., 2011; Gardner & West, 2009; West & Gardner, 2010). Altruistic behaviour evolves because of the genetic relatedness between donor and recipient due to linkage within the genome. Genetic linkage typically occurs when a set of variant gene forms are transmitted across generations as a whole because they are close to one another on a chromosome (i.e. the thread like arrangement of DNA in a cell’s nucleus). It results to indirect fitness benefits that are higher than direct fitness costs and allows altruism to evolve in a population.

There is an important reason to doubt the evolutionary stability of altruistic behaviour that relies on a green-beard complex. An ultimate explanation of altruism that relies on green-beards is vulnerable to a modifier gene (i.e. “falsebeard” gene) that emerges in the genome via mutation or recombination and subverts or replaces altruistic behaviour but retains the associated phenotypic marker that attracts fitness benefits from altruists. These modifier genes are favoured by selection because they result to higher fitness benefits and altruism is expected to have a short existence over evolutionary time.

This argument poses a challenge to ultimate explanations of cooperation that rely on emotional markers. A common research strategy is to apply insights from social evolution theory to re-examine explanations provided by evolutionary models in the social sciences (e.g. West et al., 2007; Vromen, 2017). Several evolutionary models attempt to explain human cooperation based on the presence of emotion-based phenotypic markers (e.g. Frank, 1987, 1988; Owren & Bachorowski, 2006). Using inclusive fitness analysis, it is argued that these ultimate explanation would not be evolutionarily stable (Gardner & West, 2009; Heath & Rioux, 2018; West & Gardner, 2010).

As the idea of the evolution of emotion-based social behaviours is worked out extensively by Robert Frank, the following discussion focuses on his views. In the following section I examine the structure and results of Robert Frank’s evolutionary model and the explanation it provides. Frank (1987) applies tools from evolutionary game theory to examine social behaviours and non-verbal markers. His game theoretic explanation is re-analyzed by taking into account already known ultimate explanations of cooperation. And it is argued that Frank’s model invokes a green-beard mechanism without acknowledging it (Gardner & West, 2009: p.33; West & Gardner, 2010, pp.1344, Heath & Rioux, 2018, pp.11–12). Hence, this model confronts the theoretical difficulty mentioned above. It is criticized for presenting a theoretically possible but not actually plausible ultimate explanation of human cooperation.

## Robert Frank’s evolutionary model and social behaviours

We have seen in the previous section that social evolution theorists rely on Hamilton’s work to provide a coherent framework that categorizes social behaviours based on their consequences on direct fitness (Hamilton, 1964, 1970). As long as the phenomenon to be studied is clear, the condition that allows social behaviours to evolve is straightforward: an increase in the direct or indirect fitness of the social behaviour relative to the average fitness in the population.

Inclusive fitness analysis can provide fresh insights into the ultimate explanations advanced by evolutionary models of social behaviour. In particular, Gardner and West’s analysis implies that the phenomenon explained by Frank’s model is altruistic behaviour. Moreover, this model applies a specific pleiotropy or linkage to explain the evolution of altruism. Hence, Frank’s ultimate explanation appears to be in accord with key components of the green-beard explanation presented in the previous section.

The first task is to clarify the relevant social behaviour explained in Frank’s model. Since the stability concern applies to an ultimate explanation of altruistic behaviour we cannot sweep conceptual issues under the rug and simply assume that insights from inclusive fitness analysis apply to Frank’s model.

Robert Frank engages in evolutionary modeling to examine the evolution of social behaviours. He assumes that the context of interaction is predefined in such a way as to pose a specific phenotype set (i.e. the strategies that could be applied in the game) and a payoff structure to a population of organisms. In particular, he considers a population whose members engage in a joint venture and the social interaction takes the form of a one-shot Prisoner’s Dilemma. Instead of the usual terms “defect” and “cooperate”, however, Frank’s phenotype set consists of “dishonest” and “honest” behaviours. “To be honest here means to refrain from cheating one's partner in a cooperative venture, even when cheating be cannot punished. To be dishonest means always to cheat under the same circumstances” (Frank, 1987, pp.591). To examine the evolution of these behavioural types, Frank postulates the presence of phenotypic markers or signals indicating a partner’s type. These markers are behavioural clues of emotions (e.g. respiration, posture, perspiration or facial muscle expressions) and follow a continuous probability density function.

This presentation might lead one to the conclusion that applying inclusive fitness analysis to examine Frank’s evolutionary model is fundamentally flawed because there is a difference in the phenomena explained. One can emphasize that the Hamiltonian classification of social behaviours does not refer to honesty or trust which can mean many different things in the social sciences. Moreover, Frank’s description of “honesty” (or related social behaviours like trust) does not refer to fitness effects and therefore these behaviour cannot be interpreted as representing “altruism”, “cooperation”, or any related social behaviour defined according to inclusive fitness analysis.

However, while plausible on the surface, these objections to the application of inclusive fitness analysis fail to be compelling. First, in Frank’s informal presentation of the prisoner’s dilemma, cooperation, trust and honesty are often used interchangeably (e.g. Frank, 1987, 1994). One can further note that in the more detailed presentation of the model in the book “Passions within Reason”, Frank states that the terms "defect" and "cooperate" are used to represent “dishonest” or "cheat" and “honest” or "not cheat" respectively (Frank, 1988, pp. 56). Second, it is possible to argue that Frank examines the same kind of behaviours as social evolution theory, even if he applies different labels. Although Frank’s understanding of “honesty” is not based on fitness effects, the evolution of behavioural types in evolutionary game theory relies on material payoffs that represent or are related to fitness outcomes. Hence, one can assume that in his model Frank picks out a social trait that shares the same features as cooperation in social evolution theory. Honesty and cooperation refer to a individually deleterious behaviour that benefits another organism.

So far I have left unexplored the problem of stability questioning evolutionary explanations that rely on the relation between the marker and social behaviour. Let us first look deeper into the details of Frank’s evolutionary model (Frank, 1987). In a footnote Frank writes that instead of honest (cooperative) and dishonest (defection) behaviours it is more accurate to consider two alleles a_1_ and a_2_ at a genetic locus that controls cooperation C and defection D respectively (ibid. fn. 7, pp. 595). In addition to differences in behaviour, there are also differences with respect to the heritable component μi underlying some observable human feature Si. One can further assume the presence of a second locus with two alleles, μ_H_ and μ_D_. The different combinations of the four alleles imply that there are four distinct phenotypes {CS_H_, DS_D_, CS_D_, DS_H_}. In Frank’s model, however, cooperation and defection are linked to different markers and the strategy set consists of {CS_H_, DS_D_}. It is easy to demonstrate that if cooperation C and defection D are associated with the same marker SH, and the strategy set consists of {CS_H_, DS_H_}, DS_H_ can invade the population and drive cooperation to extinction. Hence, Frank’s modelling result depends on simply assuming away the presence of green-bearded defectors DS_H_ without providing any argument as to why such a constraint in the strategy set is plausible.

To sum up, Gardner and West’s argument that Frank invokes a green-beard mechanism in the way defined by Hamilton and Dawkins does appear to follow from his evolutionary model. Although one can find different definitions of altruism, cooperation and honesty in Robert Frank’s work, it is plausible to suggest that the behaviour examined is actually altruism defined according to fitness costs and benefits. Moreover, what Gardner and West appear to analyze is Frank’s phenotype set and his assumptions about the underlying genes. Cooperation and green-beard are correlated because of a linkage between the behaviour and marker genetic loci. If the model allows for defectors to be associated with the same phenotypic marker that cooperators apply to discriminate against defectors (because of recombination or mutation), cooperation is not evolutionary stable.

In the section that follows I put aside Frank’s evolutionary model and focus on the theoretical account presented in his book. At first sight, his evolutionary explanation on the evolution of emotion-based phenotypic markers appears to be very similar to the green-beard explanation of cooperation. Under close scrutiny important differences become apparent. My main aim in the section that follows is to point out what these differences are and examine their implication for the stability problem.

## The red-beard evolutionary explanation and stability

Hamilton provided a general framework to study the evolution of cooperation, or any social behaviour: Social behaviours are categorized based on direct fitness and are favoured by natural selection if they increase inclusive fitness. One can further distinguish between ultimate explanations of cooperation on the basis of direct and indirect fitness effects. In contrast to indirect ultimate explanations that rely on genealogical kinship, green-beard explanations are not evolutionary stable unless they are based on pleiotropy.

In this section I argue that it is possible to provide a satisfactory response to the stability concern, thus vindicating the basic thrust of Frank’s account. However, in order to do so, one must examine in detail Frank’s work on human emotions and social behaviours. What I want to argue is that the standard green-beard explanation of social evolution theory and Frank’s evolutionary model does not accord with many of the key components of the evolutionary explanation presented in the rest of Frank’s work. While evolutionary models focus on fitness effects and expressed phenotype or strategies, evolutionary explanation can also refers to the emergence and evolution of psychological traits underlying social behaviours and phenotypic markers.

As a point of departure from the ultimate explanation provided by evolutionary models, one can consider that among the multiple definitions of social behaviours found in Frank’s work, some are phrased in terms of proximate causes. Specifically, Frank suggests that cooperative individuals experience emotions that commit them to their threats and promises. He writes that “a cooperator is someone who, possibly through intensive cultural conditioning, has enhanced a genetically endowed capacity to experience a moral sentiment that predisposes him to cooperate. A defector is someone who either lacks this capacity or has failed to develop it” (Frank, 1988, pp.57).

While social evolution theory and inclusive fitness analysis focuses on behaviours and fitness outcomes and do not examine proximate causal processes underlying cooperation, Frank presents an unrefined description of cooperation in terms of emotions that are part of our psychological makeup. Cooperators and defectors are understood in terms of emotions which are proximate causes of social behaviour. This understanding of cooperation is not uncommon in the social sciences (e.g. West et al., 2007). While cooperation in evolutionary theory is characterized based on fitness outcomes, the social sciences often refer to the actor’s internal processes that cause social behaviour.

Moreover, it is possible to find a second aspect of emotions in Frank’s account. Emotions such as sympathy not only motivate social behaviours like honesty, trust and cooperation in commitment problems such as the prisoner’s dilemma, but also generate phenotypic markers that others can recognize. According to Frank, emotions manifest in physical markers such as facial expression, posture, voice, eye movements that convey a person's underlying emotion. These markers involve a suite of coordinated behavioural response patterns (i.e. skeletal, facial, vocal) that express internal emotion states. This link between human expressions and emotions facilitates communication between individuals, providing information about their internal states. Individuals often lack information about others' motives, which makes it difficult to determine their behaviour and an appropriate course of action. This lack of insight of other individuals' desires, intentions or preferences was common in ancestral environments, where language as a communication device was not yet available.

What is Frank’s rationale for describing human sociality in terms of psychological mechanisms? The reason, I argue, is that in the evolutionary explanation presented in Frank’s book, proximate causes such as emotions are the target (or explanandum) of the evolutionary explanation. As argued by Frank, there is a strong relation among emotions, expressions and social behaviours. In particular, social behaviours and phenotypic markers are tied to the same internal emotional states. And it is likely that humans have psychological capacities that evolved in the ancestral environment because the behaviours they sustained conferred material benefits related to fitness. According to Frank, “behaviour is directly guided by a complex psychological reward mechanism” (Frank, 1988, pp.51) while “feelings and emotions, apparently, are the proximate causes of most behaviours” and the “task here, once again, is to explain how such sentiments might have evolved in the material world (ibid. pp.54).

Moreover, one can resist the conclusion that the phenomenon explained by Frank in his the book Passion within Reason is altruistic cooperation in the way defined by an inclusive fitness analysis. It is true that Frank states that like cooperation and honest behaviour “will be one that, by definition, requires personal sacrifice” (Frank, 1988, pp.17). But he also adds that “if character traits like honesty are observable in a person, an honest person will benefit by being able to solve important commitment problems” (ibid. p.18). One can read in these passages that if a costly social behaviour like honesty or cooperation is accompanied by a phenotypic marker, it ends up promoting an organism’s fitness. In particular, costly social behaviour and a marker end up conferring a direct fitness benefit on its bearers in comparison to self-interested behaviours with a different marker. And according to inclusive fitness analysis, such behaviours should be classified as mutually beneficial, not altruistic.

But why did emotions evolve? According to Frank, evolution would not have to build emotion-based mechanism from scratch in early human populations. Emotions related to social behaviours were initially favoured by natural selection not because of their physical manifestations, but because they contributed to the solution of problems related to human psychology. In particular, emotions may have first evolved as an impulse-control device guiding behaviour towards long-term direct fitness benefits. They allowed individuals to undergo a cost in the present and gain higher fitness benefits in the future. Next, Frank suggests, observable markers followed the activation of these emotions. Once these markers emerged, they gradually became associated in the receivers’ minds with the presence of emotions and natural selection refined them for communication purposes in one-shot interactions.

It is not my purpose to examine whether there is empirical evidence in support of Frank’s account of the origin and evolution of emotions. What is important is that this evolutionary explanation differs from simple evolutionary models’ ultimate explanation in the following way. In contrast to Frank’s model and social evolution theory that focuses on behaviours and their fitness consequences, in the evolutionary scenario described in the previous paragraph the target of the explanation or explanandum is different. What is examined is the evolution by natural selection of emotions that produce social behaviours, a marker and the recognition of the marker. More generally, selection favoured the evolution of proximate mechanisms that generate phenotypic markers and social behaviours. These proximate mechanisms have evolved because the behaviours they sustain provided, on average, fitness benefits. To distinguish this account from earlier discussions where the phenotypic marker is a green-beard, I call this the red-beard hypothesis.

Before we proceed to address the stability problem, two important clarifications are in order. First, the red-beard explanation does not a take a stance on the relation between our genetic architecture and proximate mechanisms. Despite scientific progress towards understanding human sociality, as things stand, we do not know the underlying genetics of human psychology and behaviour to any level of precision (e.g. Reuter et al, 2010; Thompson et al., 2013). It is safe to assume that some genes feed into human proximate mechanisms and social behaviours. One can further add that our proximate mechanisms are likely to be polygenic. Or that an arrangement of some genes must have been present during the evolutionary processes that lead to the formation of the proximate mechanisms that sustain social behaviours and phenotypic markers. However, which particular alleles at specified loci regulate human proximate mechanisms and behaviours remains largely unknown. Given our ignorance, if we were to advance the hypothesis that proximate mechanisms are regulated by one gene or two loci and two alleles, this dependency on a specific arrangement of genes would undermine the credibility of the red-beard explanation right from the onset.

Second, the red-beard explanation directs our attention to the possibility that the actual total causal chain that leads to social behaviour and the marker is different from the one presented by simple evolutionary models. It is possible to make this point clear if we consider what a red-beard explanation has to refer to. It will include (a) the gene(s) that shape emotion-based proximate mechanisms, (b) the two types of behaviour produced by these mechanisms (in response to prevailing environmental influences), (c) the fitness outcome of these behaviours and (d) the influence of fitness on the dynamics of gene propagation. While a red-beard explanation focuses on the evolution of proximate mechanisms, simple evolutionary models like the one presented by Frank, black-box proximate causes and target patterns of behaviours and their relation to fitness. Moreover, it is implicitly assumed that the strong association between the marker and social behaviour is either due to one gene feeding into the two behaviours or a linkage between the behaviour and marker loci. In contrast, the red-beard explanation pays attention to evolution of emotion-based proximate causes in naturally occurring interactions which simple evolutionary models largely ignore. Social emotions like guilt, shame and empathy have a genetic basis, they involve hormonal and nervous system activities and these proximate causes connect social behaviours and markers that result to fitness effects.

What about the red-beard explanation? Could it be evolutionary stable? In the critique, Gardner and West are careful to avoid citing Passions within Reasons and focus on Frank’s particular choices in model building (Gardner & West, 2009, pp.33; West & Gardner, 2010, pp.1344). In contrast to Gardner and West, a recent paper by Heath and Rioux does not distinguish between the explanation provided by the Frank’s evolutionary model and the one examined in his book (Heath & Rioux, 2018, pp.12–13).^5^ In highlighting the features of Frank’s model, they observe that according to Frank, social emotions are posited as proximate causes that explain the relation between the marker and social behaviour. They also add that it is more plausible to suggest that the falsebeard would arise in a population of cooperators with the marker, simply by losing or suppressing the capacity to cooperate (ibid. pp.11–12)^6^. What they fail to recognize is that these proximate causes are the target of the evolutionary explanation in Robert Frank’s book and they ignore them when they examine stability issues.

In moving proximate causes such as social emotions to the center stage of analysis and viewing emotion-based mechanisms as factors that are significantly changed by genetic mutations allows us to address the problem of stability. What is crucial to observe is that the stability problem arises if cooperators and defectors end up having a very similar marker.^7^ If, however, mutant genes cannot suppress cooperation without also suppressing the phenotypic marker, the stability problem does not arise.

Consider the following red-beard scenario. Suppose that genotype G feeds into an element of proximate mechanism E_M_ responsible for a social emotion E and this emotion is a common cause of two correlated joint behaviours B and S. Although correlated, there is no direct causal relationship between B and S. As an illustration, E_M_ might refer to a proximate cause or mechanism related to an emotion E such as joy or sympathy, B is a social behaviour like cooperation and S refers to a phenotypic marker such as a non-verbal expression of E. Cooperative behaviour B is correlated with a marker S only because the proximate mechanism E_M_ is a common cause of both behaviours. This can be represented by a causal diagram in the following way:

B ← E_M_ → S.

Now assume that changing E_M_ does not have other effects to the organism besides changing B and S. Moreover, the pathway through which a mutant gene influences the social behaviour B or the marker S goes through cognitive or psychological mechanisms E_M_. Suppose that a genetic change happens due to mutation that suppresses social behaviour B, as suggested by Heath and Rioux (2018). In this case, S will also change under this change of B. The reason is that changes to social behaviour B are produced by changes to the proximate emotion-based mechanism E_M_ and this mechanism is causally related to both social behaviour B and marker S. Hence, if one assumes that a mechanism E_M_ causes social behaviour B and the marker S, whenever a change of E_M_ occurs due to mutation, it will result to changes both in the social behaviour B and the marker S.

It follows that if emotions (or emotion-based proximate mechanisms) are taken into account and they causally produce social behaviour and a phenotypic marker, cooperative behaviour cannot be suppressed (or replaced by defection) without also suppressing (or losing) the phenotypic marker. In other words, those that possess or experience an emotion like sympathy that motivates cooperation will be “observably different, on the average, from those who do not” (Frank, 1988, pp.11). To the extent that psychological mechanisms of the kind that Frank proposes exist, Heath and Rioux’s argument is unconvincing. Defectors will have a different marker than cooperators and cooperators will drive them to extinction.

One might think that the red-beard hypothesis underestimates the role of natural selection to suppressing the marker or/and the social behaviour. In particular, the following two objections might come to mind. First, one might argue that natural selection would favour a mutation that suppresses both the marker and cooperation. Suppressing the whole emotion-behaviour-marker complex would save the cost of providing benefits to other cooperators. However, this objection, fails to be compelling. In the red-beard explanation, bearers of a proximate mechanism related to an emotion are fitter than non-bearers (which is equivalent to defectors without a marker) and therefore individuals do not gain by suppressing both the social behaviour and the marker. In other words, the presence of emotions attracts fitness benefits that outweigh fitness costs. If bearers of the emotion have higher fitness than non-bearers, a mutant gene suppressing the whole emotion-marker-social behaviour complex will not be favoured by natural selection.

Secondly, one might object to the red-beard hypothesis by arguing that Frank’s two-stage evolutionary account of emotions implies that natural selection resulted to a particular brain organization. Cooperators’ brain end up having two more or less independent proximate mechanisms (or one proximate mechanism with two independent sub-mechanisms), each of which is devoted to the production of a particular kind of behaviour (i.e. cooperation and the marker). This implies that natural selection could also act to change one mechanism independently of the other. In response to particular evolutionary pressures, natural selection would have favoured mutations that result in substantial changes in the proximate capacities underlying the marker production without influencing the proximate mechanism responsible for human cooperation.

While the analysis of emergence and modification of proximate mechanisms by natural selection is a complicated subject to which I cannot do justice here, such an argument is far more problematic than it first appears. The crux of this argument is the idea that two (or more) complex proximate mechanisms could have evolved independently of one another. The problem with this argument is that it does not square well with what is known from evolutionary theory. Instead of furnishing largely independent proximate mechanisms, natural selection typically modifies the proximate mechanisms that were already present in the human brain.^8^ Hence, one can retort that in the case of the red-beard hypothesis, a succession of genetic mutations over millions of years, whose phenotypes were subject to natural selection, resulted in one increasingly more complex proximate mechanism that ended up promoting non-verbal communication with others.

But even if one accepts that there are two proximate mechanisms (or a proximate mechanism with two sub-mechanisms) that underlie a particular emotion, it does not necessarily follow that natural selection can interfere with the operation of one proximate mechanism without influencing the operation of other. If the proximate mechanism involved in the production of the marker is built out of, and share parts with the proximate mechanism responsible for cooperative behaviour, a red-beard explanation would involve partly distinct mechanisms. Natural selection might change those parts shared in common and a modification to the operation of one proximate mechanism might have an impact on the other.^9^

The red-beard explanation can be readily understood by using animal signaling theory. Although the stability of signals can be maintained because of common interest, animal communication often involves a conflict of interest between signaler and receiver. In these cases, evolutionary theory suggests three main explanations why communication can be evolutionary stable (Maynard Smith & Harper, 2003; Dezecache et al, 2013; McCullough & Reed, 2016): (i) signals are indices, there are physiological, psychological, genetic or anatomical constraints that make it impossible to produce a deceptive signal; (ii) signals are handicaps, namely there are differential costs associated with the production of the marker (Zahavi, 1975); (iii) signals are stable because there are punishments costs incurred to dishonest signalers (Dezecache et al., 2013; Lachmann et al., 2001).

Given this distinction, in the red-beard explanation signals or markers are not handicaps. Nothing in the argument presented in the previous paragraphs requires that markers are costly to produce. What could be initially costly is social behaviour related to the marker, although it ends up providing fitness benefits to the organism. Moreover, behavioural responses are unaffected by retaliation and the argument presented does not rely on potential punishment costs inflicted upon falsebeards.

In the red-beard explanation phenotypic markers are indices. According to the index explanation, stability is maintained because of the presence of a tight link between an underlying internal quality and its phenotypic expression. There is a causal relation between the marker and an underlying quality and the presence or intensity of the marker is related to that quality. However, the quality signalled is not a gene or a cluster of genes (the common cause in the green-beard hypothesis), but proximate behaviour-generating emotions involved in social behaviours. What maintains cooperation is that the emergence of a defector with same marker as cooperators (i.e. “falsebeards”) is physically or psychologically difficult, because phenotypic markers are dependent on particular proximate cause of an organism.

To conclude, the red-beard and the green-beard can be held to differ with respect to what they explain. Frank’s evolutionary model and the green-beard explanation explain the evolution of social behaviour (or phenotypic markers). The red-beard explanation, however, has a different target and explains the evolution of emotion, a proximate cause that commits individuals to social behaviours and produces a phenotypic marker. In both cases, what does the explaining is natural selection and the correlation between the marker and social behaviour. The green beard explanation, however, is not stable because a mutant gene could arise that does not affect the marker but suppresses or replaces the social behaviour. In contrast, a red-beard explanation is could be stable because a mutation will change both the marker and the social behaviour.

## Conclusion

This brief examination of Robert Frank’s work has been, I think, adequate to establish the following: There are good reasons to focus on the evolution proximate causes since they often make an important difference in the stability or maintenance of social behaviours and phenotypic markers. The conventional view is that an acceptable ultimate explanation has to demonstrate that natural selection allows genes that encode for behaviours to spread, to sustain a sizable frequency in a population once they have spread and explicates why these behaviours would invade a population with alternative behaviours. While evolutionary models involve behavioural strategies and their fitness effects, we often need to examine the evolution of proximate mechanisms in complex evolutionary scenarios.

Targeting human behaviour-generating mechanisms in evolutionary explanations opens the door to new research avenues that have been previously overlooked. First, the exact strategies and payoffs and the way they change depends on the internal condition of the player(s) involved. Because many of the results depend greatly on the strategy set, taking into account human psychology and cognition and expanding the phenotype set can also change the outcome of known evolutionary dynamics. Second, because proximate psychology may be at work within individuals during the evolutionary process and can influence and modify the behaviour of conspecifics, populations starting with an interaction characterized by a game like the prisoner’s dilemma can take an evolutionary path that leads to different game. Finally, an important direction for future research is to investigate the cognitive foundations of emotion recognition. Theoretical and empirical research could investigate whether distance in space has an influence on recognition costs. Even if in emotion-mediated cooperation individuals do not incur a cost for marker recognition, there is still the question why such costless human cognitive capacities emerged, evolved and stabilized by biological evolution.

## Data Availability

Not applicable.

## Footnotes

1 Different theorists have different lists of basic emotions. For a recent review, see Keltner et al. (2019).

2 It is an open empirical question which emotions are associated with social behaviours and distinct external markers or whether there are emotional expressions that can be recognized with accuracy higher than random guessing. What complicates empirical investigation is that a given emotion may be associated with a variety of expressions, while various emotions may be related to the same expression. More experimental research is required to demonstrate if there are some emotions that are exclusively connected to few markers and particular social behaviours.

3 Proximate mechanisms can also refer to neural activity, cognitive mechanisms that regulate social learning or mental states related to beliefs and desires.

4 In line with the literature and in order to keep the analysis simple, I assume that recognition capacities are neutral in terms of fitness and do not have an influence on stability. Note, however, that in his evolutionary model Frank assumes that cooperators have to pay a fitness cost to recognize other cooperators.

5 It is confusing that in the introduction of their paper, Heath and Rioux criticize the influence of the “first generation” socio-biology in evolutionary ethics, despite the fact that their arguments against Robert Frank’s account are largely based on the inclusive fitness analysis found in the work of Gardner and West, two of the main exponents of Hamilton’s revolutionary ideas in the field of sociobiology. More recent work in evolutionary social sciences examines the evolution of proximate mechanisms and attempts to understand human cognition and psychology by “looking into the black box” (e.g. Crawford & Krebs, 2008).

6 Frank presents an alternative scenario, where defectors do not have capacity to produce the marker and the social behaviour. Due to the complexity of the underlying proximate mechanism, Frank argues that it would be difficult for defectors to acquire the same marker that cooperators use to recognize each other (Frank, 1988, 2005). Heath and Rioux do not contest this argument: it is not very plausible that a random genetic mutation will result to the all-of-a-sudden emergence of the marker in those that do not have the underlying mechanism associated with a particular emotion.

7 Cooperators and defectors do not need to end up having the same marker at all times. Provided, that the markers are different “on average”, the proximate mechanism that produces the marker and cooperation could have evolved. It is also important to note that the production of emotional markers is not all or nothing. Their clarity or distinctness is a matter of degree. It is an empirical question whether there is the threshold between a particular marker being present or being absent.

8 According to Ernst Mayr, “[T]he emergence of new structures is normally due to the acquisition of a new function by an existing structure” and “the resulting ‘new’ structure is merely a modification of a preceding” (Mayr, 1960, pp.377). More recently, evolutionary researchers such as Marcus (2006) and Anderson (2010) put forward similar ideas about proximate neural mechanisms, well supported by evolutionary arguments and empirical evidence.

9 One way of thinking about this issue at the theoretical level is to apply the distinction between “mosaic” and “connected” traits (Sterelny & Griffiths, 1999). The red-beard hypothesis is based on the idea that many emotional traits are connected traits. Hence, natural selection may not be able to modify the proximate mechanism responsible for a phenotypic marker independently of the (allegedly) distinct proximate mechanism that underlies human cooperation.
